# Low Back Pain and Its Associated Factors among Nurses in Public Hospitals of Penang, Malaysia

**DOI:** 10.3390/ijerph16214254

**Published:** 2019-11-01

**Authors:** Mohd Ismail Ibrahim, Izani Uzair Zubair, Najib Majdi Yaacob, Mohd Izmi Ahmad, Mohd Nazri Shafei

**Affiliations:** 1 Department of Community Medicine, School of Medical Sciences, Universiti Sains Malaysia, Kubang Kerian, Kota Bharu 16150, Kelantan, Malaysia; ismaildr@usm.my; 2 Penang Health State Department, 33 Pengkalan Weld, George Town, Penang 10300, Malaysia; izani_um@yahoo.com; 3 Statistic and Research Methodology Unit, School of Medical Sciences, Universiti Sains Malaysia, Kubang Kerian, Kota Bharu 16150, Kelantan, Malaysia; najibmy@usm.my; 4 Hospital Pulau Pinang, Jalan Residensi, George Town, Penang 10990, Malaysia; mohdizmi@yahoo.com.au

**Keywords:** low back pain, nurses, public hospitals, Malaysia

## Abstract

Objectives: To determine the prevalence of low back pain (LBP) and its associated factors among the nurses working in the public hospitals of Penang, Malaysia. Methods: A cross-sectional study was conducted on nurses, aged 25–60 years, who had been working for at least three months at six public hospitals of Penang. A proportionate stratified random sampling method was applied to select 1292 respondents. The Malay-validated BACKS Tool questionnaire using a 5-point Likert scale was used to obtain data. Simple and multiple logistic regression analyses were performed. Results: A total of 989 (76.5%) nurses suffered from LBP at a point of time. The factors significantly associated with LBP among the nurses included working more than seven hours [adjusted odds ratio (AOR) (95% confidence interval (CI)) 1.48 (1.06, 1.98)], twisting of the body while working [AOR (95% CI) 1.60 (1.13, 2.26)], manual handling of patients in wards [AOR (95% CI) 1.44 (1.08, 2.07)], and fatigue [AOR (95% CI) 2.63 (1.94, 3.58)]. Conclusion: The prevalence of LBP among the nurses in the public hospitals of Penang was relatively high. The factors predicting LBP included working more than seven hours a day, twisting of the body while working, manual handling, and fatigue. The findings from this study may better enable policymakers to devote resources to minimize low back pain among nurses. The nurses should be encouraged to comply with safe working procedures.

## 1. Introduction

Low back pain (LBP) is a common, disabling, and burdensome disorder affecting people worldwide. Lumbar strain and sprains caused by stretch injury to the tendons, ligaments, or muscles of the lower back region lead to acute or chronic LBP. Back injuries can occur because of overuse, improper use, or trauma [1]. Straining and stretching of the muscles can occur from a twisting, bending, or lifting procedure, lifting a heavy object, or overstretching [2]. 

People who have LBP suffer for a certain duration of time, and after recovery, they eventually return to normal activities. Nevertheless, some of them develop chronic pain and few of them suffer from a disability. It was reported that, one year after the first episode of back pain, 62% of the people still have pain and 16% of those who were unable to work are still not working [3]. 

Many researchers studied the problems associated with LBP among workers including drivers, office workers, school teachers, and nurses. Of the commercial vehicle drivers in Malaysia, 60.4% were suffering from LBP. On the contrary, 37% of the office workers in Malaysian public universities developed LBP [4]. Meanwhile, a study in a hospital in Sarawak, Malaysia, found that staff nurses had the highest prevalence of LBP (38.8%), followed by community nurses (19.0%) and doctors (13.7%), and assistant medical officers had the lowest prevalence (5.6%) [5]. 

Nurses were found to have a six-time higher prevalence of back injuries in comparison to other health professionals [6]. It would lead to an increase in work absenteeism and cost of occupational disability among them [7,8]. Nurses who had LBP also required a median of seven days to recuperate [9]. In European countries, an eight-year longitudinal study in a university hospital in Switzerland revealed that the prevalence of LBP among nurses varies from 73% to 76% [10]. Meanwhile, the prevalence of work-related back injuries among the nurses at a teaching hospital in China was reported to be 56% [11]. On top of that, the one-year prevalence of LBP was reported to be about 86% and 67% among ambulance nurses and those working in orthopaedic departments, respectively [12,13]. In Jordan, the one-year prevalence among nurses was reported to be 79% [14].

In terms of the impact of LBP on workers, a study involving 187 countries pointed out that it was the major cause of disability and absence from work [14,15]. A few studies have demonstrated that physical and mental demands may bring nurses to terminate their jobs [16,17]. Persistent LBP can decrease the quality of life among workers and affect them psychologically [18,19]. 

LBP not only affects the people suffering from it but also the organizations with which they are associated. It is due to their absenteeism and presenteeism at work. A study in the United Kingdom highlighted that rising absenteeism in the latter half of the 20th century and rising bills for incapacity benefits due to back pain are shared by the United Kingdom and other developed countries [20]. In addition, the quality of patient care is also disrupted that indirectly increases the burden of work on other nurses in the same ward.

LBP has a major economic impact worldwide in terms of the cost that is bearable by organizations [21,22,23]. In the United States, it was reported that patients with musculoskeletal conditions incur a total annual medical care cost of approximately $77 billion [24]. In addition, it was found that LBP can be a financial burden because of the high cost of workers’ compensation, insurance to be paid to injured workers, recruitment or training costs, recovery from LBP being time consuming, and return-to-work rehabilitation [25].

LBP among nurses can be contributed by few factors such as their sociodemographic, work-related, psychosocial, and lifestyle factors such as smoking and improper exercise, as supported by Genaidy et al. [26]. Hence, the present study was designed to determine the prevalence of LBP and its associated factors among nurses working in public hospitals. It would provide local data that are considered useful for intervention strategies to improve LBP among these nurses. 

## 2. Materials and Methods

### 2.1. Study Design and Respondents Selection

A cross-sectional study was conducted at six public hospitals of Penang, Malaysia, which consisted of three hospitals with medical specialists and three hospitals without medical specialists. Table 1 provides a list of the selected hospitals with the number of nurses. The study was conducted from 1 April, 2016, to 31 July, 2016. Nurses, aged between 25 and 60 years, who had been working for at least 3 months were included in the study. Those who were pregnant, suffering from chronic LBP, and working as community or dental nurses were excluded from the study. 

The largest and feasible sample size was calculated using a single proportion formula, with the proportion of nurses with LBP as 41% using a previous study [27]. The precision was set at 0.03, and using type 1 error as 5%, the required sample size was 1292 after considering a non-response rate of 20%. A proportionate stratified random sampling method was applied to select 1292 respondents from a total of 2499 nurses available at the time of the study. The nurses were divided according to the types of hospitals (district and general), which formed the strata. Within each type of hospitals, we performed simple random sampling to select the nurses to be included in the study. 

### 2.2. Research Tools

The BACKS Tool questionnaire that was granted permission by the researcher of the Universiti Kebangsaan Malaysia was used for this study. It was a Malay-validated questionnaire developed to assess work-related chronic LBP in Malaysia [28]. This self-administered questionnaire consisted of sociodemographic characteristics, work environment, pain visual scale, and presence of back pain in a year. It was scored on the basis of the total score of a number of questions from the first three sections. The score was used to categorize the respondents into work-related and nonwork-related back pain. It was reported that the questionnaire had a sensitivity of 62.7% and specificity of 94.5% for the detection of work-related back pain [28].

The job demand subscale (from the work environment domain) consisted of two factors, namely, physical and psychological demands. Each item was rated on a 5-point Likert scale (1: Strongly disagree, 2: Disagree, 3: Unsure, 4: Agree, and 5: Strongly agree). Test–retest was used to perform the reliability test of the research instrument within the study population. The composite reliability of the factors using Raykov’s rhos in both models was good. Although it was observed that Raykov’s rho was 0.680 for the physical demand in the two-factor model, the value was acceptable because it was slightly less than the cutoff value of 0.7. The rest of the Rayko’s rho values were greater than the cutoff value of 0.7 [29].

### 2.3. Data Collection and Statistical Analyses

A self-administered BACKS Tool questionnaire was applied to obtain data from the respondents. All the respondents were divided into small groups and called to attend the data collection session according to their allocated time. They were briefed about the study before providing their written consent to participate.

The nurses in Malaysia are subjected to work either in rotating shifts or during the day (nonshift). Those who work in a rotating shift schedule are considered shift workers. Work schedule that is practiced by nurses in public hospitals involves a fast-forward rotating shift of 2 days of morning shift, followed by 2 days of evening shift and 2 days of night shift. Then, they get 2 days off from work. The work starts at 7:00 for the morning shift, 14:00 for the evening shift, and 21:00 for the night shift. Hence, the maximum number of hours that nurses work per day is 10 hours, which is during the night shift. There is no difference in terms of working duration between both types of hospitals (with or without medical specialists). In contrast, those who practice day work (from 8:00 to 17:00) are considered nonshift workers. They work 5 days a week from Sunday to Thursday, with 2 days off. 

In the present study, the respondents with pain, muscle tension, or stiffness localized below the costal margin and above the inferior gluteal folds, with or without sciatica, were grouped as “having LBP.” On the basis of the BACKS Tool questionnaire, only those who answered that they engaged in any physical activity for at least three times a week and a minimum of 30 minutes per session were grouped as “engaged in physical activity.” Otherwise, they were grouped as “not engaged in physical activity.”

Data entry and analyses were performed using Statistical Package for Social Sciences (SPSS, version 22; IBM Corp., Armonk, NY, USA). Data were checked, explored, and cleaned. Simple and multiple logistic regression analyses were performed to obtain the factors associated with LBP among the nurses. The selection of variables was performed using manual, forward, and backward selection methods. The independent variables included sociodemographic, work-related, psychological, and lifestyle factors. The preliminary main-effect model was obtained after comparing the model using backward and forward likelihood ratio methods. Multicollinearity was tested using the correlation matrix. All possible two-way interactions were checked. The fitness of the model was assessed by the Hosmer–Lemeshow goodness-of-fit test, classification table, and area under the receiver operating characteristics (ROC) curve. The level of significance was set at a *p*-value of less than 0.05.

### 2.4. Ethical Consideration

The present study received ethical approval from the Research Ethics Committee (Human) of Universiti Sains Malaysia (USM/JEPeM/15090308), and the National Medical Research Review Register of Malaysia (NMRR-15-1668-27637). The approval to use the BACKS Tool was obtained from the Centre for Collaborative Innovation, Universiti Kebangsaan Malaysia. The study was funded by the Research University (Individual) Grant by the Universiti Sains Malaysia. The confidentiality of the data was maintained throughout the study, with only the researchers having access.

## 3. Results

The response rate of the present study was 100% as we got good cooperation and collaboration from the hospitals’ top management and Penang state health department. Most of them (59.5%) were less than 30 years of age and the majority were female (96.2%). Table 2 provides the sociodemographic characteristics of the nurses working in the public hospitals of Penang, who participated in the study. Most of them did not smoke [1279 (99%)] and some of them [627 (48.8%)] claimed to be engaged in physical activities. 

Table 3 presents the occupational characteristics of the respondents. The nurses who worked in hospitals with medical specialists [1152 (89.2%)] were more in number than those in hospitals without medical specialists [140 (10.8%)]. Most of them worked for less than five years [684 (53.0%)]. 

Table 4 provides the psychological factors affecting the respondents in the present study. Most of the nurses were satisfied with their work [1051 (81.3%)]. Meanwhile, 1051 (95.3%) of them responded that they felt fatigued during working hours. A total of 1231 (95.3%) agreed that they got good cooperation from their colleagues, and 1058 (81.9%) agreed to receive good cooperation from their supervisors and employers.

It was found that 989 (76.5%) of the respondents suffered from LBP at a point of time (within the past year). Using multiple logistic regression analysis, the factors that were significantly associated with LBP among the nurses were working longer than seven hours [adjusted odds ratio (AOR) (95% confidence interval (CI)) 1.48 (1.06, 1.98)], twisting of the body while working [AOR (95% CI) 1.60 (1.13, 2.26)], manual handling of patients in wards [AOR (95% CI) 1.44 (1.08, 2.07)], and fatigue [AOR (95% CI) 2.63 (1.94, 3.58)]. Table 5 shows the summary of the findings.

There was no significant interaction and multicollinearity. The Hosmer–Lemeshow test (chi-square: 3.676, df = 5, *p* = 0.597) suggested that the model was fit. The overall percentage of the classification table was 77.6%, which meant that the model could accurately predict 77.6% of the cases. The area under the ROC curve was 0.655 [95% CI (0.618, 0.691)]. 

## 4. Discussion

Nurses play a major role in managing patients, and the nature of their work exposes them to back pain. In the present study, the one-year prevalence of LBP among the nurses working in public hospitals was 74.8%. The finding is almost similar to the study among the nurses in Switzerland [10], which ranged from 73% to 76%. It is also in line with the studies in Kuwait, Tunisia, and Nepal [30,31,32]. However, the prevalence was lower in developed countries such as Canada, Ireland, and Japan [33,34,35]. The prevalence of LBP among nurses may differ between developed and developing countries, within countries, and across the region. The differences of the findings could be due to the difference in the research methodology, questionnaire used, history of back pain among nurses, and self-report of the disease having a pattern of recurrence and reduction. On top of that, the application of back hygiene policy and workplace interventions also play a major role in the differences. Despite the modernization of the health care system, the studies in Taiwan and South Africa reported that the lifetime prevalence of LBP among nurses working in hospitals was 82% and 84%, respectively [36]. It indicates that regardless of the modernization status of the country, pain could occur if there were no preventive measures taken while working.

The present study shows that working hours play a significant role in the development of LBP. It was found that those who work more than seven hours a day were at risk of LBP as compared to others. It can be due to the repetitive exposure to excessive work, such as lifting a heavy object, which leads to injury to the lower back region. Furthermore, sometimes they need to work a few hours more covering for their colleagues on emergency leave or when there is lack of manpower. This is in line with a study in Jeddah, Saudi Arabia, which describes that nurses who worked more than 10 hours a day had an increased risk of LBP compared to those who did not [37]. In addition, Shieh et al. [38] described that the risk of LBP increases by 35% for every additional daily working hour. As a result, it was suggested that the work schedule of nurses needs to be relooked into, as it plays a significant role in the development of back pain [39].

Nurses usually worked longer than their normal schedule because of the shortage of registered nurses [40]. With a big ratio between nurses and patients, the risks of making errors were significantly increased. Fatigue is one of the symptoms that arises because of work exhaustion, which can contribute to accidents and injuries at the workplace. On top of that, it was reported that the incidence of LBP is increased with an increase in working hours [41]. In addition, a study found that prolonged working hours exposed nurses to repeated manual handling and, thus, sustained the accumulation of wears and tears of the back muscles [42].

Another important factor in the development of LBP is manual handling performed by nurses. Many nurses in Malaysia are still required to manually lift patients from one place or one position to another. All nurses in Malaysia are required to use small aids like sliding sheets to move patients in the hospitals. However, the present study found a significant association between manual handling and LBP. The repetitive work exposes them to injuries, especially in the lumbar area. It is caused by the kinetic imbalance and pressure exerted on this area. The repeated load application by lifting a patient may result in cumulative fatigue and reduction in the stress-bearing capacity of nurses [43]. 

Apart from lifting patients, nurses often conduct patient handling by bending their waists and maintaining an uncomfortable posture toward the opposite side of the bed or chair. This position increases the risk of back pain [44]. This is supported by a study in the Netherlands and Iran, which found that the bending position increases the risk of back pain [45,46]. However, in the present study, the bending position was not significantly associated with the development of LBP. This difference could be due to the recall bias of the respondents.

Nurses frequently need to twist their bodies while handling patients. In the present study, this has a significant association with LBP. This is in line with the studies in India and Brazil, which found that twisting of the body was associated with LBP [47,48]. On top of that, back muscle fatigue is found to be strongly associated with decreased postural stability especially in standing position. The present study revealed that there was a significant association between fatigue and LBP. This can be explained by the negative influence of fatigue on the muscle receptors and thereby on proprioception [49]. 

Among the limitations of the present study were the involvement of nurses from a different level of sociodemographic characteristics, working experiences, different lifestyle, or psychological background, which creates heterogeneity. Thus, a proper random selection based on the inclusion and exclusion criteria was used in order to minimize the selection bias and control the findings of the study. Apart from that, most of the variables used in the analyses of the present study were from the self-reported assessment which were collected using self-administered questionnaires. The issue concerning response bias (also known as survey bias) has been recognized as a potential threat to the internal validity of studies utilizing questionnaires as tool for data collection. Response bias occurs when respondents tend to answer questions untruthfully or misleadingly due to any reason such as social acceptability. This bias is a known key problem in the data collection process of most observational epidemiological research designs. Similarly, in the present study, we recognized that the response bias might have subsequently led to an inaccurate estimation of prevalence and association. Nevertheless, a self-reporting data collection method can help provide valuable responses pertaining to the perspective of nurses.

Our approach to statistical analysis has one limitation in which a weighted statistical analysis might offer more accurate findings, as it takes into consideration the sampling weight within each stratum. Weighted statistical analysis is widely used in survey research, where a complex design sampling method is used for the selection of study participants. In this study, a weighted statistical analysis was not performed, as we did not regard the data as survey data. In addition, since the prevalence of LBP was high in the present study, the risk might have been overestimated with the use of odds ratio in the analyses.

It is recommended for the Nursing Division, Ministry of Health of Malaysia, to design an effective intervention to improve the condition. Many studies have shown that the combination of treatment and health education, as well as correct back exercises, is effective in treating mild to moderate LBP. The Ministry of Health may need to evaluate the handling policy to ensure all healthcare workers have a good working practice. In addition, the current policy needs to be evaluated to ensure the current problem is addressed appropriately and action is taken against those who do not comply with it. 

On top of that, an interesting approach to the problem of lower back pain among nurses is the family-friendly hospital concept. In this approach, family members are encouraged to assist nurses in taking care of patients. They should be allowed to perform basic procedures such as assisting nurses in lifting or mobilizing patients during bedside management. To minimize any implication, they need to be trained on the basic procedure so that it could be done in a correct manner. However, as far as we can determine, such an approach has only been evaluated in a single study in India where the researchers found that the approach gives encouraging results [50].

## 5. Conclusions

The prevalence of LBP among the nurses in the public hospitals of Penang was relatively high, which could be attributed to the nature of their work, compared to other occupational sectors. Among the factors that predict LBP among nurses include working more than seven hours a day, twisting of the body while working, manual handling, and fatigue. There is a need for the Ministry of Health to design an effective intervention to improve the condition. Many studies have shown that the combination of treatment and health education, as well as correct back exercises, is effective in treating mild to moderate LBP. The nurses should also be encouraged to comply with the safe working procedures available. 

## Figures and Tables

**Table 1:** List of nurses in the public hospitals of Penang, Malaysia (as of August 2015).

Name of Hospital	No. of Nurses
Hospital Pulau Pinang	1403
Hospital Seberang Jaya	580
Hospital Bukit Mertajam	243
Hospital Kepala Batas	148
Hospital Sungai Bakap	68
Hospital Balik Pulau	57
**Total**	**2499**

**Table 2:** Sociodemographic characteristics of the respondents.

Variables	All Respondents (*n* = 1292)	Respondents with LBP (*n* = 989)	Respondents without LBP (*n* = 303)
	*n* (%)	*n* (%)	*n* (%)
**Age (years)**			
Less than 30	769 (59.5)	600 (60.7)	169 (55.8)
30–40	383 (29.6)	291 (29.4)	92 (30.3)
More than 40	140 (10.9)	98 (9.9)	42 (13.9)
**Body mass index**			
Underweight	113 (8.7)	113 (11.4)	21 (6.9)
Normal	653 (50.5)	469 (47.5)	163 (53.8)
Overweight	368 (28.5)	287 (29.0)	81 (26.7)
Obese	158 (12.3)	120 (12.1)	38 (12.6)
**Gender**			
Male	49 (3.8)	35 (3.5)	14 (4.6)
Female	1243 (96.2)	954 (96.5)	289 (95.4)
**Marital status**			
Single	420 (32.6)	315 (31.9)	105 (34.7)
Married	872 (67.4)	674 (68.1)	198 (65.3)
**Highest education**			
Diploma	1041 (80.6)	786 (79.5)	255 (84.2)
Degree or higher	251 (19.4)	203 (20.5)	48 (15.8)
**No. of children**			
0	596 (46.2)	459 (46.4)	137 (45.2)
1–3	600 (46.4)	462 (46.7)	138 (45.5)
4 and more	96 (7.4)	68 (6.9)	28 (9.3)

**Table 3:** Occupational characteristics of the respondents.

Variables	All Respondents (*n* = 1292)	Respondents with LBP (*n* = 989)	Respondents without LBP (*n* = 303)
	*n* (%)	*n* (%)	*n* (%)
**Type of hospital**			
With specialist	1152 (89.2)	885 (89.5)	267 (88.1)
Without specialist	140 (10.8)	104 (10.5)	36 (11.9)
**Department**			
Medical base	908 (70.3)	705 (71.3)	203 (67.0)
Surgical base	384 (29.7)	284 (28.7)	100 (33.0)
**Duration of working as a nurse (years)**			
0–5	684 (53.0)	526 (53.2)	149 (49.2)
5–10	288 (22.3)	223 (22.6)	65 (21.4)
10–15	163 (12.6)	120 (12.1)	43 (14.2)
More than 15	157 (12.1)	120 (12.1)	46 (15.2)
**Working hours/day (hours)**			
1–7	277 (21.4)	193 (19.5)	84 (27.7)
More than 7	1015 (78.6)	796 (80.5)	219 (72.3)
**Shift work**			
Yes	1071 (82.9)	819 (82.8)	252 (83.2)
No	221 (17.1)	170 (17.2)	51 (16.8)
**Manual handling**			
Yes	1115 (86.3)	874 (88.4)	241 (79.5)
No	177 (13.7)	115 (11.6)	62 (20.5)
**Bending of the body**			
Yes	1233 (95.2)	948 (95.9)	282 (93.1)
No	62 (4.8)	41 (4.1)	21 (6.9)
**Twisting of the body**			
Yes	1082 (83.7)	851 (86.1)	231 (76.2)
No	210 (16.3)	138 (13.9)	72 (23.8)
**Lifting heavy object**			
Yes	1194 (92.4)	930 (94.0)	264 (87.1)
No	98 (7.6)	59 (6.0)	39 (12.9)
**Estimated lifting weight (kg)**			
Less than 10	281 (21.7)	195 (19.7)	86 (28.4)
More than or equal to 10	1011 (78.3)	794 (80.3)	217 (71.6)
**Lifting technique**			
Use body	1131 (87.5)	864 (87.4)	267 (88.1)
Use instrument	161 (12.5)	125 (12.6)	36 (11.9)
**Carry heavy objects**			
Yes	600 (46.4)	479 (48.4)	121 (39.9)
No	692 (53.6)	510 (51.6)	182 (60.1)
**Mobilize patient on bed**			
Yes	1098 (85.0)	851 (86.0)	247 (81.5)
No	194 (15.0)	138 (14.0)	56 (18.5)
**Mobilize patient from bed to chair**			
Yes	1043 (80.7)	810 (81.9)	233 (68.0)
No	249 (19.3)	179 (18.1)	97 (32.0)
**Assist patient from bed to toilet**			
Yes	934 (72.3)	728 (73.6)	206 (68.0)
No	358 (27.7)	261 (26.4)	97 (32.0)

**Table 4:** Psychological factors of the respondents.

Variables	All Respondents (*n* = 1292)	Respondents with LBP (*n* = 989)	Respondents without LBP (*n* = 303)
	*n* (%)	*n* (%)	*n* (%)
**Headache**			
Yes	653 (50.5)	539 (54.5)	114 (37.6)
No	639 (49.5)	450 (45.5)	189 (62.4)
**Stress**			
Yes	697 (53.9)	786 (79.5)	262 (86.5)
No	595 (46.1)	203 (20.5)	41 (13.5)
**Low mood**			
Yes	548 (42.4)	44.3 (44.8)	105 (34.7)
No	744 (57.6)	546 (55.2)	99 (32.7)
**Fatigue**			
Yes	1051 (95.3)	847 (85.6)	204 (93.4)
No	241 (4.7)	142 (14.4)	99 (32.6)
**Work satisfaction**			
Yes	1051 (81.3)	786 (79.5)	262 (86.5)
No	241 (18.7)	203 (20.5)	41 (13.5)
**Support from colleague**			
Yes	1231 (95.3)	948 (95.9)	283 (93.4)
No	61 (4.7)	41 (4.1)	20 (6.6)
**Support from supervisor/employer**			
Yes	1058 (81.9)	798 (80.7)	260 (85.8)
No	234 (18.1)	191 (19.3)	43 (14.2)

**Table 5:** Factors associated with low back pain (LBP) among the nurses in the public hospitals of Penang, using multiple logistic regression analysis (*n* = 1292).

Variables	Crude OR (95% CI)	Adjusted OR (95% CI)
**Age (years)**		
Less than 30	1	
30–40	0.89 (0.67, 1.19)	
More than 40	0.66 (0.44, 0.98)	
**Gender**		
Female	1.00	
Male	1.32 (0.70, 2.49)	
**Marital status**		
Single	1.00	
Married	1.14 (0.87, 1.49)	
**No. of children**		
None	1.00	
Between 1 and 3	0.10 (0.76, 1.31)	
More than 3	0.73 (0.45, 1.17)	
**Body mass index**		
Underweight	1.00	
Normal	0.69 (0.41, 1.14)	
Overweight	0.90 (0.47, 1.38)	
Obese	0.72 (0.40, 1.13)	
**Type of working hospital**		
Hospital with specialist	1.00	
Hospital without specialist	1.15 (0.77, 1.72)	
**Department**		
Medical base	1.00	
Surgical base	1.22 (0.93, 1.61)	
**Duration of working as nurse (years)**		
0–5	1.00	
5–10	0.97 (0.70, 1.35)	
10–15	0.79 (0.53, 1.17)	
>15	0.74 (0.50, 1.09)	
**Working hours/day (hours)**		
1–7	1.00	1.00
More than 7	1.58 (1.18, 2.13)	1.48 (1.06, 1.98)
**Shift work**		
Yes	1.00	
No	0.98 (0.69, 1.37)	
**Carry heavy object**		
No	1.00	
Yes	1.41 (1.09, 1.84)	
**Twisting of the body**		
No	1.00	1.00
Yes	1.92 (1.40, 2.65)	1.60 (1.13, 2.26)
**Manual handling**		
No	1.00	1.00
Yes	1.96 (1.40, 2.75)	1.44 (0.99, 2.07)
**Stress**		
No	1.00	
Yes	2.10 (1.61, 2.73)	
**Fatigue**		
No	1.00	1.00
Yes	2.90 (2.15, 3.90)	2.63 (1.94, 3.58)

Backward and forward likelihood ratio methods were used to obtain the preliminary main-effect model as both methods retain similar number of variables. Classification table: 77.6%. Hosmer–Lemeshow test: Chi-square: 3.676, df = 5, *p* = 0.597. Area under the ROC curve: 0.655 [95% CI (0.618, 0.691)]; OR = odds ratio; CI = confidence interval.
